# Light‐chain amyloid myopathy isolated to skeletal muscles: A case report

**DOI:** 10.1002/ccr3.3310

**Published:** 2020-09-08

**Authors:** Toshihiro Matsukawa, Katsuki Eguchi, Ichizo Nishino, Kohei Okada, Kazuo Oshimi, Takuto Miyagishima

**Affiliations:** ^1^ Department of Internal Medicine/Hematology Kushiro Rosai Hospital Kushiro Japan; ^2^ Department of Neurology Kushiro Rosai Hospital Kushiro Japan; ^3^ Department of Neuromuscular Research National Institute of Neuroscience Tokyo Japan

**Keywords:** amyloidosis, isolated amyloid myopathy, light‐chain

## Abstract

Isolated amyloidosis, especially of amyloid light‐chain type, is an infrequent disease. Systemic chemotherapy for light‐chain amyloidosis isolated to skeletal muscles plays a key role to reduce clonal plasma cells producing aberrant immunoglobulin.

## Graphical abstract

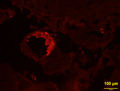

## 1 INTRODUCTION

Muscle specimens of a man revealed light‐chain amyloid myopathy isolated to skeletal muscles and the bone marrow aspiration unveiled an abnormal population of plasma cells. He received chemotherapy and autologous stem cell transplantation, which led to long‐term survival without disease progression.

Amyloidosis is a rare disease caused by amyloid deposits, which is an insoluble fibrous proteins consisting of beta‐sheet structure^1^ in tissues and organs such as the heart and kidneys, damaging their functions. Amyloidosis is divided into different groups; amyloid light‐chain (AL) amyloidosis, AA amyloidosis (known as secondary amyloidosis, resulted in chronic inflammatory or infectious disease), dialysis‐related amyloidosis, familial (hereditary) amyloidosis, wild‐type transthyretin (senile systemic) amyloidosis, and organ‐specific (localized) amyloidosis.^2^ AL amyloidosis is the most common form that results from immunoglobulin light‐chain production by abnormal plasma cells subsequently leading to multiple myeloma (MM), which is characterized by an abnormal proliferation of plasma cells and the production of M protein. According to the International Myeloma Working Group, myeloma defining events (MDEs) include hypercalcemia, renal failure, anemia, and bone lesions.^2^ Systemic chemotherapy is required in case of symptomatic MM, where MDEs or myeloid‐defining biomarkers are present. Amyloid myopathy usually occurs as one of the various symptoms of systemic amyloidosis. Isolated amyloid myopathy is a much rarer disease compared to systemic amyloidosis. Prayson reported that isolated amyloid myopathy accounted for no more than 0.004% (16 cases) of all muscle specimens, which were obtained from patients with suspected myopathy.^3^ We, herein, report a rare case of AL amyloid myopathy isolated to skeletal muscles treated with systemic chemotherapy and stem cell transplantation (SCT) achieving a good response.

## 2 CASE REPORT

**Figure 1:**
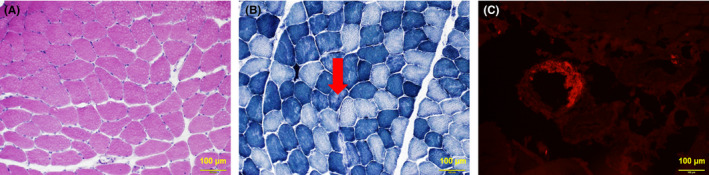
Representative pathological images of a rectus femoris muscle biopsy. On hematoxylin and eosin (A), fiber size variation is mild. On nicotinamide adenine dinucleotide tetrazolium reductase (B), intermyofibrillar network is mildly disorganized in some fibers. On Congo red (C), amyloid is deposited in the area surrounding muscle fibers. Scale bars: 100 μm

**Figure 2:**
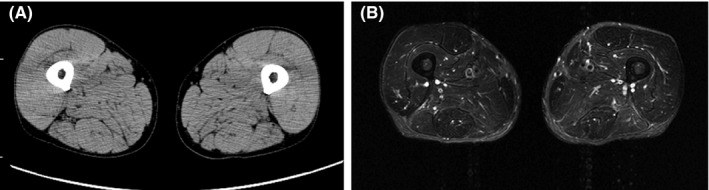
Leg muscle imaging. Computerized tomography (CT) (A), T2‐weighted short‐tau inversion recovery magnetic resonance imaging (MRI) (B). CT and MRI scans did not show any abnormalities

A 55‐year‐old man developed pain, tingling, and numbness in both upper and lower parts of his legs with gradual exacerbation. He presented to an orthopedic clinic four months later, but was not diagnosed with any condition. Because of the progressive nature of his symptoms, he visited our hospital for further evaluation. He complained of worsening symptoms on both legs. He did not report recent weight loss, dysphasia, or diarrhea. His family history, past medical history, and drug history were unremarkable. He was a coal miner, and his other social history was unremarkable. His blood pressure was 150/92 mm Hg, his temperature was 36.6℃, his pulse was 71/min, and O_2_ saturation was 98% at room air. Macroglossia was not seen. His neck was supple. The results of his cardiovascular examination were normal, the lungs were clear on bilateral auscultation, and the results of his abdominal examination were unremarkable with no hepatosplenomegaly. He showed muscle weakness in both upper and lower legs. Neurological tests showed no abnormalities. Manual muscle strength testing showed a grade 4/5 strength of both upper and lower legs based on the Medical Research Council scale. The complete blood counts were normal (hemoglobin: 14.6 g/L, mean cell volume: 94.3 fL, white blood cell count: 9.4 × 10^9^/L with 68.3% of neutrophils and 23.3% of lymphocytes, and platelets: 296 × 10^9^/L); inflammatory markers were also normal (C reactive protein: 0.04 mg/L; normal range: 0.0‐0.3 mg/dL, erythrocyte sedimentation rate: 10 mm/h; normal range: 0‐10 mm/h). Liver enzymes were slightly elevated; lactate dehydrogenase (349 U/L; normal range: 113‐270 U/L), alkaline phosphatase levels (45 U/L; normal range: 12‐34 U/L), and alanine transaminase levels (88 U/L; normal range: 9‐46 U/L). The total bilirubin level (0.5 μmol/L; normal range: 0.2‐1.2 mg/dL) as well as total protein level (8.0 g/dL; normal range: 6.7‐8.3 g/dL) were normal; however, the albumin level (3.1 g/L; normal range; 3.9‐4.9 g/L) was slightly reduced. The serum creatine kinase (CK, 7143 U/L; normal range: 46‐201 U/L) and aldolase levels (10.0 U/L; normal range: 1.0‐7.5 U/L) were over the normal upper limits. The brain natriuretic peptide level was within normal limit (11.7 pg/mL; normal level: ≤18.4 pg/mL). Antibodies against aminoacyl tRNA synthetase (ARS), Sjögren's (SSA/SSB), and histidyl tRNA synthetase (JO‐1) as well as neutrophil cytoplasm antibodies (pANCA/cANCA) were all negative. These results excluded collagenous disease and autoimmune disease. We performed an electrocardiogram and nerve conduction tests, the results of which were normal. Electromyography showed normal discharge at rest. Early motor unit potentials were within the normal range in all muscles including the bilateral biceps femoris muscles and the rectus femoris muscles. Although myopathies or neuropathies were unlikely according to the electromyography, his clinical symptoms and elevated CK/aldolase levels still suggested them. The patient was then referred for muscle biopsy. On muscle biopsy from the right rectus femoris, hematoxylin and eosin staining showed mild variation in muscle fiber size without any necrotic or regenerating fibers (Figure 1A). No mononuclear cell infiltration was seen. On nicotinamide adenine dinucleotide tetrazolium reductase, intermyofibrillar network is nonspecifically disorganized in some fibers (Figure 1B). On Congo red, amyloid was deposited in the area surrounding muscle fibers (Figure 1C). The pathological findings led us to suggest amyloidosis. Quantitative serum immunoglobulins analyses demonstrated elevated IgG levels (3381 mg/dL, normal range: 870‐1700 mg/dL) and decreased IgA (33 mg/dL, normal range: 110‐410 mg/dL), and IgM levels (18 mg/dL, normal range: 35‐220 mg/dL). Serum immunoelectrophoresis showed monoclonal gammopathy (IgG‐λ). The serum kappa/lambda ratio was 0.03 (normal range: 0.26‐1.65). The level of serum kappa and lambda free light‐chains were 7.9 mg/L (normal range: 3.3‐19.4 mg/L) and 266.0 mg/L (normal range: 5.7‐26.3 mg/L), respectively. Because amyloidosis or MM was suspected because of elevated IgG levels and the abnormal serum‐free light‐chain ratio, bone marrow aspiration, and biopsy were performed. Bone marrow aspirate smear showed 18% of plasma cells infiltration. Flow cytometry analysis showed an abnormal population of plasma cells that accounted for 18.5% of normal cells, most of which were positive for surface CD54, CD56, CD138, and cytoplasmic λ light‐chain. Fluorescence in situ hybridization test detected translocation of t(4;14) but not t(14;16) or del(17p). The bone marrow biopsy specimen was negative to Congo red staining. A whole‐body computed tomography (CT) revealed no evidence of lymph node swelling, bone lesions, or any other abnormalities. Furthermore, a CT scan of his legs revealed no dystrophy or myodemia (Figure 2A). No abnormalities were observed on the T2‐weighed magnetic resonance imaging (MRI) (Figure 2B). Echocardiogram demonstrated an ejection fraction of 75.4% without myocardial hypertrophy including interventricular septal thickness or any other abnormalities. Abdominal fat pad aspiration and biopsies from his gastric and rectal mucosa revealed no evidence of infiltration of amyloid deposits. These findings led to a diagnosis of AL amyloid myopathy isolated to skeletal muscles because the patient had abnormal plasma cell proliferation with a lack of MDEs or amyloid deposits in other areas.

**Figure 3:**
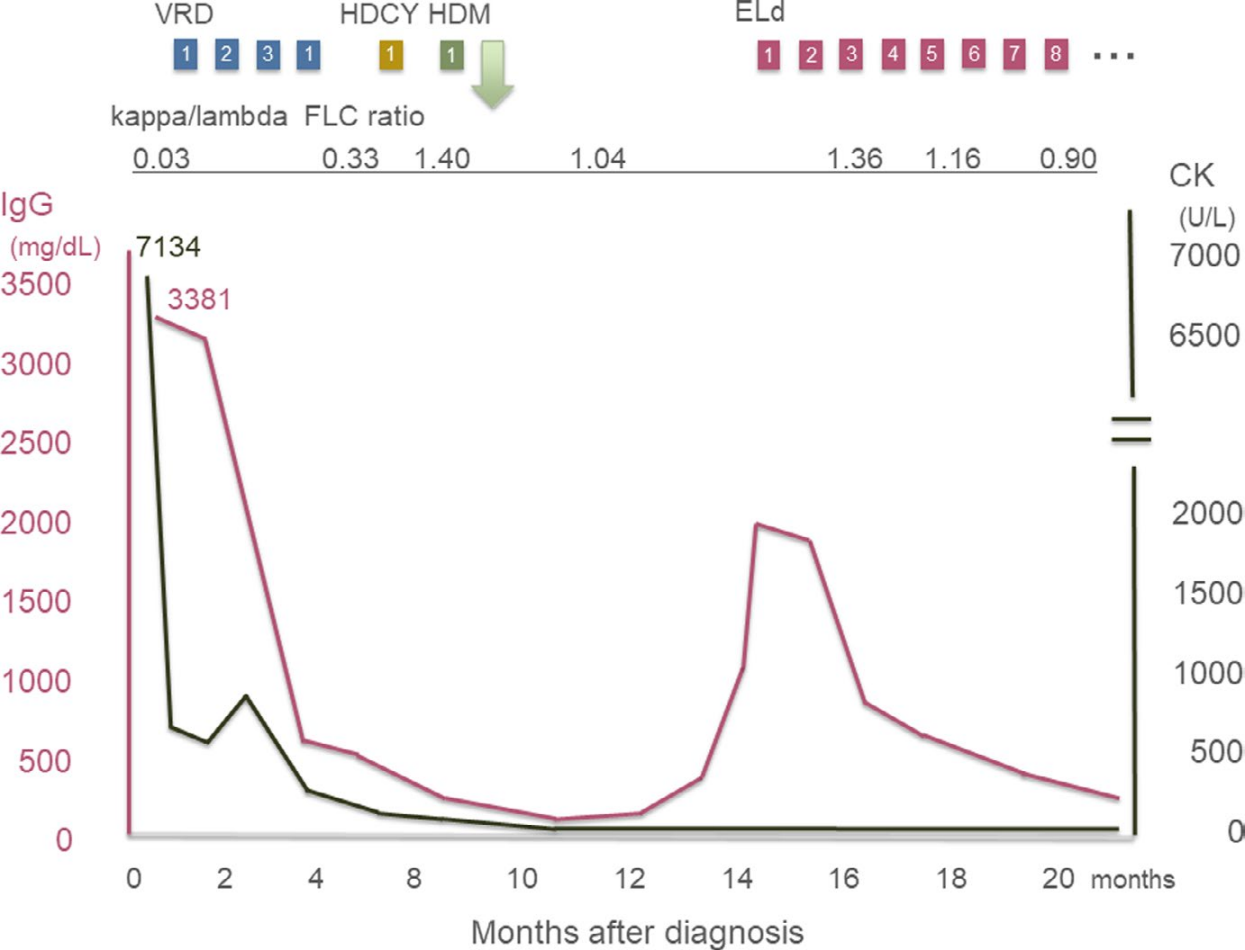
Clinical course from the diagnosis. The green arrow shows auto peripheral blood stem cell transplantation. The number in a colored block represents the number of cycles of chemotherapy. VRD, bortezomib, lenalidomide, and dexamethasone; HDCY, high‐dose cyclophosphamide; HDM, high‐dose melphalan; ELd, elotuzumab, lenalidomide, and dexamethasone; CK, creatine kinase; FLC, free light‐chain

As shown in Figure 3, he was administered bortezomib (an inhibitor of the 26S proteasome), lenalidomide (an immunomodulatory drug), and dexamethasone (a corticosteroid) (VRD). After four courses of VRD, high‐dose melphalan (an alkylating agent) administration followed by autologous peripheral blood SCT was performed. He decided not to receive maintenance therapy after SCT. His leg muscle weakness gradually exacerbated, and finally, he had to rely on a wheelchair occasionally during SCT. Because his serum paraprotein levels gradually increased five months after he received SCT, the patient was administered elotuzumab (a humanized IgG1 monoclonal antibody targeting SLAMF7), lenalidomide, and dexamethasone, which resulted in a very good partial response. He did not indicate any signs of progression over four years, although his muscle weakness was not alleviated.

## 3 DISCUSSION

The present case showed isolated AL amyloid myopathy, which sporadically occurred in the leg muscles. Bone marrow aspiration indicated a gene rearrangement and increment of monoclonal plasma cells although the patient did not have any MDEs. AL amyloids are reported to be prone to deposit specific organs according to light‐chain repertoires^4^, ^5^. Previous research has shown that only 2.7% of isolated AL amyloidosis such as cardiac, renal, and lymph node involvement were reported in patients who had amyloid deposition in abdominal fat pad and/or bone marrow, resulting in progression to systemic amyloidosis requiring systemic chemotherapy.^6^

Some reports mentioned amyloid deposition affects the kidneys, heart, gastrointestinal tract, nervous system, peripheral nerves in systemic amyloidosis, except the skeletal muscles.^7^, ^8^, ^9^ Shimazaki et al concluded that systemic amyloidosis infiltrated the skeletal muscles in 0.67% of cases.^7^ As discussed above, there were only 0.004% of amyloid myopathy cases in suspected myopathies.^3^ These studies indicate that amyloid myopathy is rarely detected even if myopathy is suspected. Likewise, Liewluck et al reported 14 isolated cases (27%) in 52 amyloid myopathy patients.^10^ The remaining 38 cases included 32 systemic AL amyloidosis, while the 14 isolated cases evidently failed to detect systemic AL amyloid. Furthermore, the report suggested that isolated amyloid myopathy cases presented asymptomatic hyperCKemia and were young‐onset without dysphagia and weight loss. These symptoms are likely to be well compatible with our case, except being asymptomatic. Median CK levels of isolated amyloid myopathies increased 10.9‐29.0‐fold compared to systemic amyloid myopathy.^10^ In support of this report, over half of systemic amyloidosis patients have increased CK levels.^6^ Our case was younger‐onset and showed 35.5 times higher CK levels than the upper normal limit.

To our knowledge, there are only a few reports of isolated AL amyloidosis cases, especially AL amyloid myopathy.^11^, ^12^, ^13^, ^14^ One case of isolated AL amyloid myopathy required colchicine, but the patient died as a result of cardiac arrest after four months of treatment.^13^ A large cohort study in approximately 1200 patients showed systemic AL amyloidosis with over 10% bone marrow plasma cells (BMPCs) or MDEs and shortened median overall survival (16.2 months for the first group and 10.6 for the second group) compared to cases without these features whose median overall survival was 46 months.^15^ Systemic cases generally have adverse outcomes without treatment, and the prognosis of isolated cases remains controversial ranging from a few months to several years. Given this fact, it can be assumed that short survivors with isolated amyloidosis actually had amyloid deposition in other parts of the body such as the heart without being detected, or the outcome depended on the therapies they received. Our patient continues to live without any signs of amyloidosis besides limb muscle weakness. There has been a reduction in his levels of abnormal plasma cells and amylogenic production from them in response to serial chemotherapy and SCT.

The aim of systemic AL amyloidosis treatment is the cessation of abnormal plasma cell propagation and excess light‐chain production. Little is known about how to treat isolated amyloidosis, although our patient was started on bortezomib for the treatment of systemic AL amyloidosis. Bortezomib rapidly acts with good response in AL amyloidosis, and the National Comprehensive Cancer Network and the European Myeloma Network recommend high‐dose melphalan followed by SCT for eligible patients with systemic AL amyloidosis ^16^, ^17^ because SCT prolongs overall survival and improves complete remission rate compared to high‐dose chemotherapy.^18^, ^19^ Melphalan and bortezomib have been used as one of the key drugs for amyloidosis.^20^ Moreover, a combination therapy of bortezomib in addition to dexamethasone with melphalan and/or cyclophosphamide provides a better prognosis for amyloidosis.^21^, ^22^, ^23^, ^24^, ^25^, ^26^ A recent prospective study showed that upfront bortezomib usage led to a longer‐duration hematological response.^27^ Daratumumab, an anti‐CD38 monoclonal antibody for MM, combined with a chemotherapy agent such as bortezomib also led to improved treatment response and survival outcomes in AL amyloidosis.^28^, ^29^ In summary, our case was very rare and suggests that isolated AL amyloid myopathy requires chemotherapy and/or SCT like systemic AL amyloidosis.

The patient and his family provided their consent for the publication of the present case.

## CONFLICT OF INTEREST

None declared.

## AUTHOR CONTRIBUTIONS

TM: designed the study, collected data, attended the patient, and wrote the manuscript. KE: collected data and attended the patient. IN: wrote parts of the draft on the pathological images and obtained the pathological images for the current case; KO: collected data and attended the patient. KO and TM: attended the patient. All authors edited, reviewed, and approved the final version of the manuscript.

## ETHICAL APPROVAL

The written informed consent to publication was obtained.
